# Water Supply Interruptions and Suspected Cholera Incidence: A Time-Series Regression in the Democratic Republic of the Congo

**DOI:** 10.1371/journal.pmed.1001893

**Published:** 2015-10-27

**Authors:** Aurélie Jeandron, Jaime Mufitini Saidi, Alois Kapama, Manu Burhole, Freddy Birembano, Thierry Vandevelde, Antonio Gasparrini, Ben Armstrong, Sandy Cairncross, Jeroen H. J. Ensink

**Affiliations:** 1 Environmental Health Group, Department of Disease Control, Faculty of Infectious and Tropical Diseases, London School of Hygiene & Tropical Medicine, London, United Kingdom; 2 Ministère de la Santé Publique, Division Provinciale de la Santé Publique, District Sanitaire d’Uvira, Uvira, Sud-Kivu, République Démocratique du Congo; 3 Ministère de la Santé Publique, Division Provinciale de la Santé Publique, Bukavu, Sud-Kivu, République Démocratique du Congo; 4 Fondation Veolia, Nanterre, France; 5 Department of Medical Statistics, Faculty of Epidemiology and Population Health, London School of Hygiene & Tropical Medicine, London, United Kingdom; 6 Department of Social and Environmental Research, Faculty of Public Health and Policy, London School of Hygiene & Tropical Medicine, London, United Kingdom; University of North Carolina at Chapel Hill, UNITED STATES

## Abstract

**Background:**

The eastern provinces of the Democratic Republic of the Congo have been identified as endemic areas for cholera transmission, and despite continuous control efforts, they continue to experience regular cholera outbreaks that occasionally spread to the rest of the country. In a region where access to improved water sources is particularly poor, the question of which improvements in water access should be prioritized to address cholera transmission remains unresolved. This study aimed at investigating the temporal association between water supply interruptions and Cholera Treatment Centre (CTC) admissions in a medium-sized town.

**Methods and Findings:**

Time-series patterns of daily incidence of suspected cholera cases admitted to the Cholera Treatment Centre in Uvira in South Kivu Province between 2009 and 2014 were examined in relation to the daily variations in volume of water supplied by the town water treatment plant. Quasi-poisson regression and distributed lag nonlinear models up to 12 d were used, adjusting for daily precipitation rates, day of the week, and seasonal variations. A total of 5,745 patients over 5 y of age with acute watery diarrhoea symptoms were admitted to the CTC over the study period of 1,946 d. Following a day without tap water supply, the suspected cholera incidence rate increased on average by 155% over the next 12 d, corresponding to a rate ratio of 2.55 (95% CI: 1.54–4.24), compared to the incidence experienced after a day with optimal production (defined as the 95th percentile—4,794 m^3^). Suspected cholera cases attributable to a suboptimal tap water supply reached 23.2% of total admissions (95% CI 11.4%–33.2%). Although generally reporting less admissions to the CTC, neighbourhoods with a higher consumption of tap water were more affected by water supply interruptions, with a rate ratio of 3.71 (95% CI: 1.91–7.20) and an attributable fraction of cases of 31.4% (95% CI: 17.3%–42.5%). The analysis did not suggest any association between levels of residual chlorine in the water fed to the distribution network and suspected cholera incidence. Laboratory confirmation of cholera was not available for this analysis.

**Conclusions:**

A clear association is observed between reduced availability of tap water and increased incidence of suspected cholera in the entire town of Uvira in Eastern Democratic Republic of the Congo. Even though access to piped water supplies is low in Uvira, improving the reliability of tap water supply may substantially reduce the incidence of suspected cholera, in particular in neighbourhoods having a higher access to tap water. These results argue in favour of water supply investments that focus on the delivery of a reliable and sustainable water supply, and not only on point-of-use water quality improvements, as is often seen during cholera outbreaks.

## Editors' Summary

**Background:**

Every year, outbreaks (epidemics) of cholera—a bacterial gut infection caused by *Vibrio cholerae*—make 2–3 million people ill and kill about 100,000 people. Because people get cholera by drinking water or eating foods contaminated by the feces of infected individuals, cholera outbreaks usually occur in places with poor sanitation and poor access to clean water, such as slums and refugee camps. Natural disasters that disrupt sanitation and water systems can also trigger cholera outbreaks. Most people who become infected with *V*. *cholerae* have no or mild symptoms, but they may shed bacteria in their feces for up to two weeks. Other infected individuals develop severe diarrhea, producing profuse watery feces. With prompt treatment—replacement of lost fluids and salts by drinking an oral rehydration fluid or by injection of rehydration fluid into a vein in the worst cases—less than 1% of infected individuals die. Without treatment, people with severe cholera can die from dehydration within hours of developing symptoms.

**Why Was This Study Done?:**

Cholera control strategies generally focus on the provision of safe drinking water through treatment of water supplies at source or at point of use. But could addressing the reliability of household water sources in regions with poor access to piped treated water and other improved water supplies help to control cholera? A water source that provides an unpredictable amount of clean water is likely to increase the incidence of cholera (the number of new cases in a population in a given time period) through causing the occasional use of unsafe water sources, unsafe water storage, and limited water availability for handwashing. In this time-series regression (a statistical method for analyzing data collected over time), the researchers investigate the temporal association between water supply interruptions and admissions to the cholera treatment center in Uvira, a town in a province of the Democratic Republic of the Congo where cholera is endemic (always present). Although 80% of Uvira’s population regularly or occasionally uses the town’s chlorinated water supply, only a quarter of the population has access to a water tap in their yard or dwelling, and the water supply is intermittent and unreliable.

**What Did the Researchers Do and Find?:**

The researchers examined data on the daily number of people over 5 y old admitted to Uvira’s cholera treatment center with diarrhea in relation to daily variations in the volume of water supplied by the town’s water treatment plant between 2009 and 2014. In the 12 d following a day without tap water, the incidence of suspected cholera increased by 155%. That is, compared to the 12 d following a day with optimal water production, there were 2.55 times as many cases of cholera in the 12 d following a water supply interruption. Moreover, 23.2% of suspected cholera cases over the study period were potentially attributable to a suboptimal tap water supply. Compared to neighborhoods with low tap water consumption, the incidence of suspected cholera was lower in neighborhoods with high tap water consumption. However, in these neighborhoods, water supply interruptions increased the incidence of suspected cholera in the following 12 d nearly 4-fold and 31.4% of suspected cholera cases were attributable to water supply interruptions. Finally, there was no association between the levels of residual chlorine in water fed to the distribution network and the incidence of suspected cholera.

**What Do These Findings Mean?:**

These findings show an association between reduced availability of tap water and increased incidence of suspected cholera in an urban setting in the Democratic Republic of the Congo where cholera is endemic. Thus, improving the reliability of the water supply might substantially reduce the incidence of cholera, particularly in neighborhoods with greater access to tap water. Some aspects of the study design may affect the accuracy of these findings. For example, because laboratory confirmation of cholera was not available, some of the patients admitted to the cholera treatment center with diarrhea may not have had cholera. However, these findings argue in favor of focusing on the delivery of a reliable and sustainable water supply in cholera-endemic areas as a strategy for cholera control and, more generally, highlight the public-health importance of providing access to reliable tap water supplies, a concept now included by the WHO/UNICEF Joint Monitoring Program (an official body tasked with monitoring global water access) in its definition of a safely managed water source.

**Additional Information:**

This list of resources contains links that can be accessed when viewing the PDF on a device or via the online version of the article at http://dx.doi.org/10.1371/journal.pmed.1001893.

- This study is further discussed in a *PLOS Medicine* Perspective by Clarissa Brocklehurst and Tom Slaymaker
- The World Health Organization (WHO) provides information about cholera and about diarrhea; it also provides information on water, sanitation and health and on drinking water quality (in several languages)
- The US Centers for Disease Control and Prevention provides information about cholera (including information on cholera control strategies) for the public, medical professionals and travelers
- The UK National Health Service (NHS) Choices website provides information about cholera
- MedlinePlus provides links to further resources about cholera (in English and Spanish)
- The not-for-profit organization Médecins Sans Frontières (MSF) is tackling several cholera outbreaks around the world; its website includes a description of the ongoing humanitarian crisis in the Democratic Republic of the Congo
- Personal stories about cholera in the Democratic Republic of the Congo are available from UNICEF, a not-for-profit organization that protects the rights of children and young people around the world
- The WHO/UNICEF Joint Monitoring Programme for Water Supply and Sanitation (JMP) monitors global access to safe drinking water and improved sanitation; the JMP Update Report 2015 is available online
- The non-governmental organization Practical Action provides information and approaches and technologies for improving urban water supplies

## Introduction

In 2012, the Democratic Republic of the Congo (DRC) reported more than 28% of all reported cholera cases in Africa, and 27% of cholera-related deaths globally [1]. The Great Lakes region and particularly Eastern DRC have been identified as a stable transmission focus for cholera, where cases of cholera have been reported every year since 1978 [2,3]. South-Kivu province, in particular, is considered as an endemic area, reporting cases nearly continuously since 2000 [4]. Although detailed data for this particular area is scarce, access to safe water in DRC in 2015 is generally poor, with 52% of the population using improved water sources, and only 8% of the population having access to piped water on premises [5].

Cholera has been predominantly linked with contaminated water ever since John Snow removed the pump handle from the London Broad Street pump in 1854. However, more recent research confirmed the role of direct human-to-human transmission already suggested by John Snow as an important route in the 1850s [6,7]. This more direct pathway has been suggested as an explanation for the explosive nature of cholera outbreaks, along with hyperinfectivity of cholera organisms when freshly shed by an infected individual [8].

Cholera control strategies, especially during outbreaks, generally focus on the provision of clean drinking water and the removal of potential contamination of that water by means of water treatment at source, or at point of use [4,9]. They also commonly include activities that promote personal, food, and household hygiene but more rarely address the issue of the amount of water available to a household and the reliability of the water source, even though those factors will impact water collection, storage, and hygiene practices and, as a result, the microbial quality of the water at point of use [10,11].

Particularly in endemic areas, an unreliable water supply that provides an unpredictable amount of tap water to households is therefore likely to increase cholera incidence through the occasional use of unsafe sources of water, unsafe water storage, and restricted personal and household hygiene behaviours. Water supply interruptions may also lead to contamination of the water in the piped network due to low or negative pressure and ingress of pathogens. There is a growing body of evidence on the impact of inconsistent access and use of improved water sources on the microbial quality of water at point of use [12]. However, when searching for published evidence of the impact of water, sanitation, and hygiene interventions during cholera epidemics, we found only a single study in which piped water supply was implemented in a cholera-affected community; it showed a 65% reduction in endemic cholera incidence, though the study suffered from major design flaws [13]. A recent review on water supply interventions impact on diarrhoeal diseases found only limited evidence on the impact of continuous and safe quality piped water supply on diarrhoeal diseases; a single study reported an estimated 73% reduction in the risk of diarrhoeal diseases compared to noncontinuous piped water supply [14]. The degree to which the unreliability of clean water supply affects cholera incidence, especially in endemic areas, has not been previously investigated.

Using data collected in a middle-sized town in DRC, our study explores the association between the daily volume of chlorinated tap water distributed to households and admissions of suspected cholera cases to the local Cholera Treatment Centre (CTC) with a time-series regression analysis.

## Methods

### Study Area

The city of Uvira is located in South Kivu Province in Eastern DRC on the shores of Lake Tanganyika, and had an estimated population of 205,000 people in 2012. The town is spread over nearly 10 km along Lake Tanganyika at an altitude of 800 m above sea level and is crossed by three rivers. Uvira has a moderate tropical climate with an average temperature of 26°C throughout the year.

The population relies on surface water sources (the lake and rivers) and on the water supply managed by the national water agency (Regideso), which provides chlorinated water through a sparse network of approximately 2,800 private and shared taps across town. It is estimated that 80% of the population regularly or occasionally uses this municipal water supply, and of that group, only a quarter have access to a tap in their yard or dwelling. Unreliable power supply from the local grid, limited resources for generator use, irregular supply of chemicals for water treatment (i.e., chlorine, aluminium sulphate, lime), frequent downtime for routine maintenance, and occasional equipment failure all result in an intermittent and unreliable supply of tap water.

Between 2004 and 2013, at least one case of suspected cholera has been reported weekly by the Uvira district health office, and a Cholera Treatment Centre (CTC) in Uvira district hospital has been set up to admit all patients with severe acute diarrhoea. Patients are treated for dehydration and administered broad spectrum antibiotics. However, in the absence of adequate local laboratory facilities, regular confirmation of cholera is not conducted, except occasionally at the onset of suspected cholera outbreaks. Admission and treatment at the CTC is free of charge for all patients.

### Suspected Cholera Case and Water Supply Data

Daily admissions to the CTC during the period of January 1, 2009, to April 30, 2014, were extracted from the database held by the district health office since 2009, which is updated weekly from the paper register used by clinical staff at the CTC. Patients admitted at the CTC are recorded along with their neighbourhood of residence, and patients residing outside of Uvira municipality were excluded from the analysis. In order to conform to the WHO suspected cholera case definition in endemic areas, patients aged 5 y or under were excluded [9].

Daily volume of water supplied for the same time period was collected from the register held at the Regideso water treatment plant in Uvira, in which river water drawn upstream of town is treated by sedimentation/flocculation and chlorination before being fed to a single 1,600 m^3^ reservoir supplying the town gravity distribution system. Volumes of water supplied over 24 h were measured with a flowmeter placed at the only water treatment station output, where the levels of residual chlorine in the supplied water were also measured by means of N,N diethyl-p-phenylene diamine (DPD) tablets and the daily average (of over two or more measurements) recorded in the same register.

Although the piped water system serves, in theory, all areas of Uvira municipality, the 184 neighbourhoods of Uvira were classified as having a higher or lower tap water consumption based on an estimated monthly average volume of tap water available per person. This was calculated for each neighbourhood on the basis of the volume of water billed in February 2012 to 2,630 Regideso taps (each of which is geographically referenced and allocated to a particular neighbourhood by global positioning system [GPS]) divided by the estimated neighbourhood population. Based on actual tap metering, this estimate accounts for the large variability between taps in use (shared or private) and water availability that is dependent on the tap location on the distribution network.

Daily precipitation rates in mm/h estimates averaged for Uvira area at a 0.25 degree by 0.25 degree spatial resolution were obtained from NASA Tropical Rainfall Measuring Mission (TRMM) 3B42-v7, through the Giovanni online data system, developed and maintained by the NASA Goddard Earth Sciences Data and Information Services Center (NASA GES DISC). Daily variations of temperature are small in Uvira, and no complete daily temperature record for the region was identified. Temperature was therefore not included as a confounder in the analysis.

### Statistical Methods

The relationship between the daily number of admissions at the CTC and the daily volume of water supplied by the Regideso was examined using generalized linear Poisson regression models allowing for overdispersion [15]. To account for seasonality and long-term trends in potential unmeasured confounders, a cubic spline of date (12 degrees of freedom [df] per year) was included in the model, as a more flexible alternative than including month-in-year terms. Terms for the day of the week (indicators) and daily precipitation rates (linear) were also added. An autoregressive term was introduced to the model in order to control for a significant temporal auto-correlation at days 1 and 2.

The relationship between water supply level and suspected cholera incidence was modelled using the framework of distributed lag nonlinear models (DLNMs). These models allow the net effect of an exposure to be computed as the sum of contributions at different lags, through the definition of a lag-response curve in addition to the exposure-response relationship [16]. DLNMs were originally developed for investigating temperature-health associations [17].

Informed by a recent review of incubation periods for cholera and delays due to storage capacity in the water production system, we considered lags up to 12 d and assumed that effects, if any, rose from none at lag 0 [18].

A linear exposure-response function scaled against the 95th percentile of volume (subsequently referred to as optimal production) was considered for association between volume and admissions. The delay in effect of water volume on admissions (modelled as a lag-response curve) was constrained to follow a smooth quadratic curve.

The overall cumulative effect of volume produced on CTC admissions over 12 d was computed by summing effects over the whole lag period. The association is reported as relative risk (RR).

The number and proportion of suspected cholera cases attributable to water production lower than the 95th percentile of volume were estimated from the model, as defined by Gasparrini and Leone [19]. These calculations assume that a fraction (RR-1)/RR of suspected cholera cases on any given day is attributable to the exposure to a suboptimal water supply during the 12 previous days modelled to give a relative risk, and are computed for the entire study duration. The figures are interpreted as the number of suspected cholera cases attributable to suboptimal water production during the study period, which are potentially preventable if the volume of water supplied was maintained above the optimal production level.

We explored the potential heterogeneity of effect in neighbourhoods with a higher or a lower tap water consumption. The median value of tap water invoiced across neighbourhood in February 2012 was 2.8 l per person and per day and this was taken as cut-off value for stratification of neighbourhoods into higher and lower tap water consumption. A regression model was fitted separately to each series of higher and lower consumption, based on the same specifications used in the main analysis. These models also included the number of admissions in the other area as an additional term, in order to account for potential inter-neighbourhoods transmission.

The sensitivity of the estimates to the modelling assumptions outlined above was tested by the comparison with models specified with different choices. The model parameters tested included the number of df of the spline function of date, the shape of the lag-response function, inclusion of an impact on day 0 and inclusion of a term for chlorine residual levels.

All analyses were performed in the R environment using the package dlnm [20]. The R code for reproducing the main results is included in S3 Text. The full code is available from the corresponding author on request.

### Study Design and Ethics

Local authorities and international non-governmental organisations (NGO) involved in cholera control activities in Uvira observed that major cholera epidemics often seemed to follow water supply interruptions. In order to investigate this possible association, the authors retrospectively collected relevant data from the local water agency Regideso and from Uvira CTC, and the reliability, size, and completeness of the dataset was deemed sufficient to perform a time-regression analysis. No formal prospective analysis plan was submitted.

This study was approved by the Observational Committee of the London School of Hygiene & Tropical Medicine Research Ethics committee (reference 7745). The Committee waived informed consent from participants as the data were anonymously collected and analysed.

## Results

**Fig 1:**
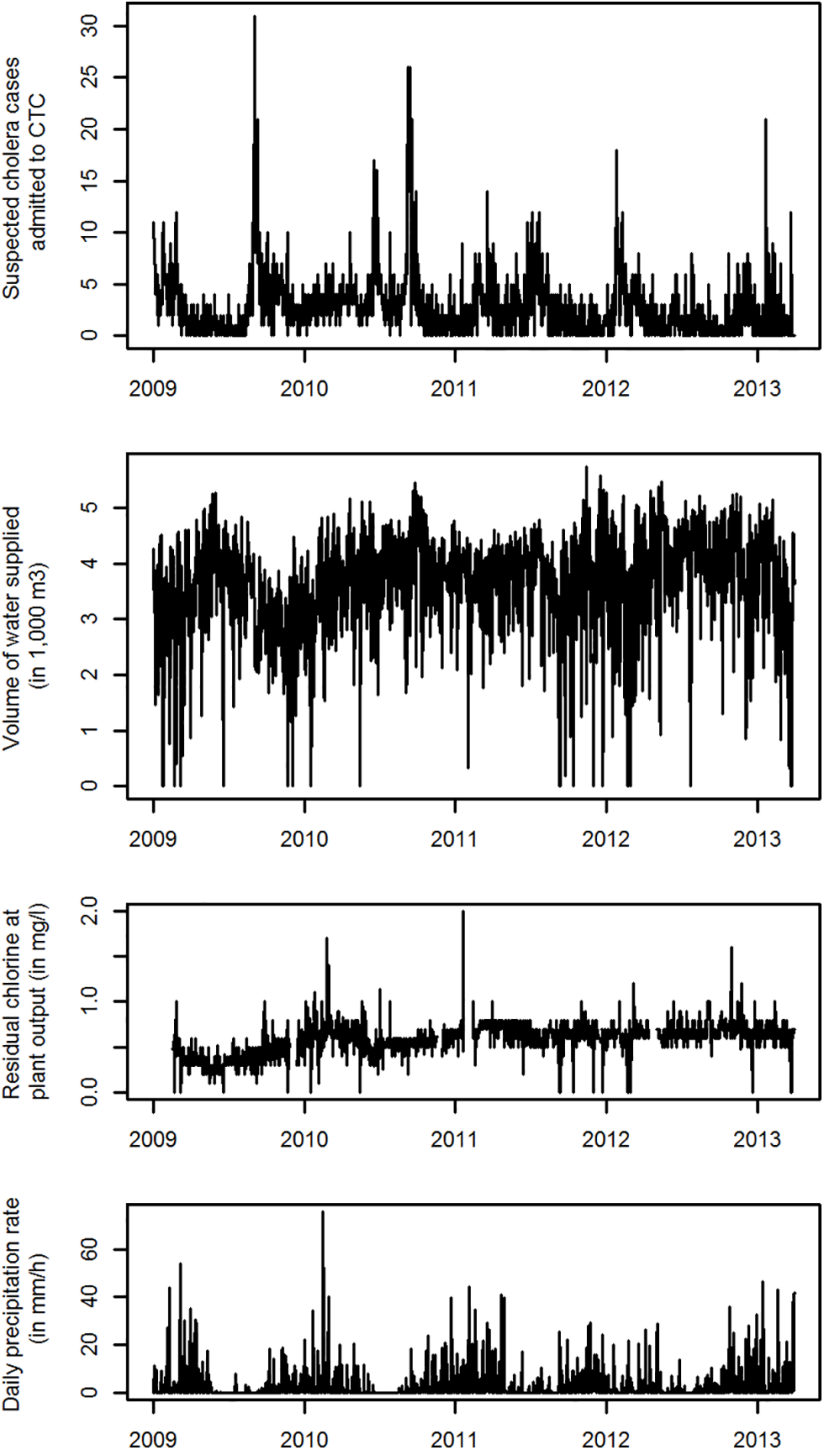
Daily time series between January 1, 2009, and April 30, 2014, for admissions at the CTC, volume of water supplied, level of residual chlorine in produced water and precipitation rate in Uvira, DRC.

Between January 1, 2009, and April 30, 2014, a total of 5,745 patients aged over 5 y were admitted to the CTC, with daily admissions ranging between 0 and 32. Daily volume of water supplied to the city ranged from 0 to 5,748 m^3^ with a 95th percentile (“optimal” production) of 4,794 m^3^, while the level of residual chlorine ranged from 0.1 to 1.7 mg/l (Table 1 and Fig 1).

**Table 1:** Distribution of admissions to CTC, volume of tap water supplied, and levels of residual chlorine in the water between January 1, 2009, and April 30, 2014, in Uvira, DRC.

	Range	Median	Interquartile range	95th percentile	Number of records (% missing)
Daily admissions to CTC (all town)	0 to 32 patients	2	1–4	8	1,946 (0%)
Daily admissions to CTC (neighbourhoods with tap water consumption ≥2.8 l/day/person)	0 to 18 patients	1	0–2	5	1,946 (0%)
Daily admissions to CTC (neighbourhoods with tap water consumption <2.8 l/day/person)	0 to 18 patients	1	0–2	6	1,946 (0%)
Daily volume of water supplied	0 to 5,748 m^3^	3,741	3,072–4,264	4,794	1,944 (0.1%)
Daily average of residual chlorine in water supplied	0.1 to 1.7 mg/l	0.60	0.50–0.70	0.80	1,839 (5.5%)
Daily precipitation rate	0 to 70.7 mm/h	0.4	0–2.6	14.2	1,944 (0.1%)

### Association of Volume of Water Supplied with CTC Admissions

**Fig 2:**
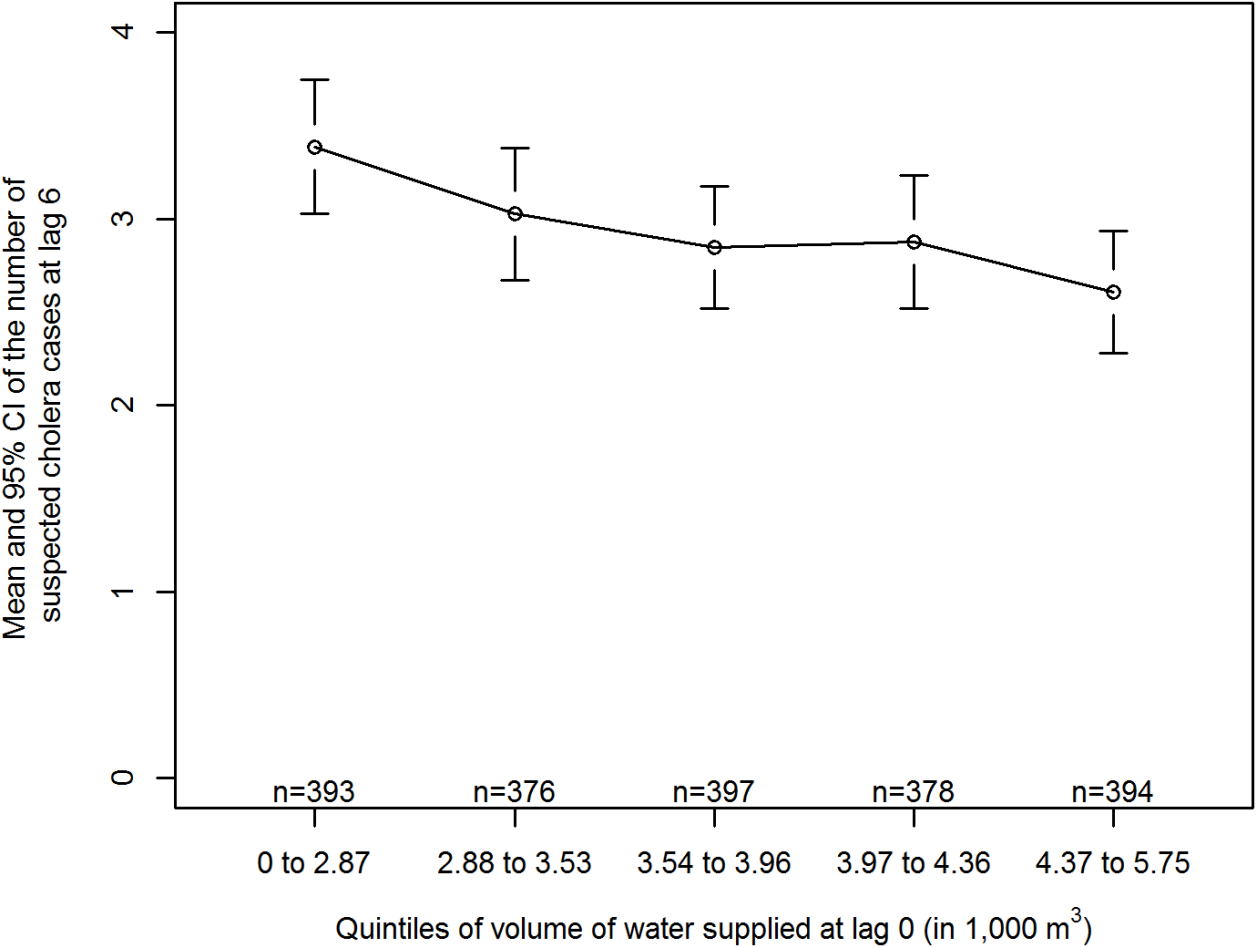
Mean incidence of suspected cholera at lag 6 d by quintile of volume of water supplied at lag 0 day. n: number of days.

**Fig 3:**
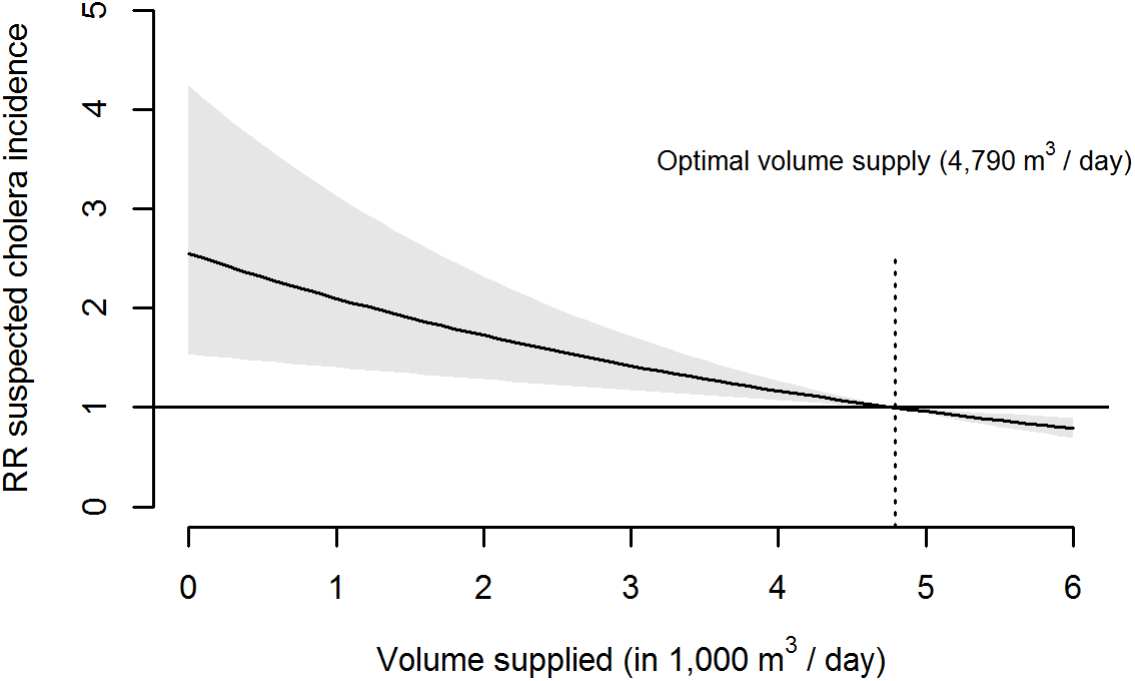
Twelve-day cumulative association of volume of tap water supplied with suspected cholera incidence in Uvira. Shaded area: 95% CI for relative risk (RR)–model predictors: daily volume of water supplied, cubic spline of date, day of the week, precipitation rate of the day and number of CTC admissions on the previous 12 d.

A crude group analysis shows a decreasing mean number of admissions at lag 6 d with an increasing volume of water supplied (Fig 2). Fig 3 displays the overall cumulative exposure-response relationship between volume of water supplied and incidence of suspected cholera as modelled with the DLNM time-series regression. When the water treatment plant produces no water, the model predicts an increase of 155% in the number of suspected cholera cases within the next 12 d, corresponding to an RR of 2.55 (95%CI: 1.54–4.24), compared to the optimal production level (4,794 m^3^).

**Fig 4:**
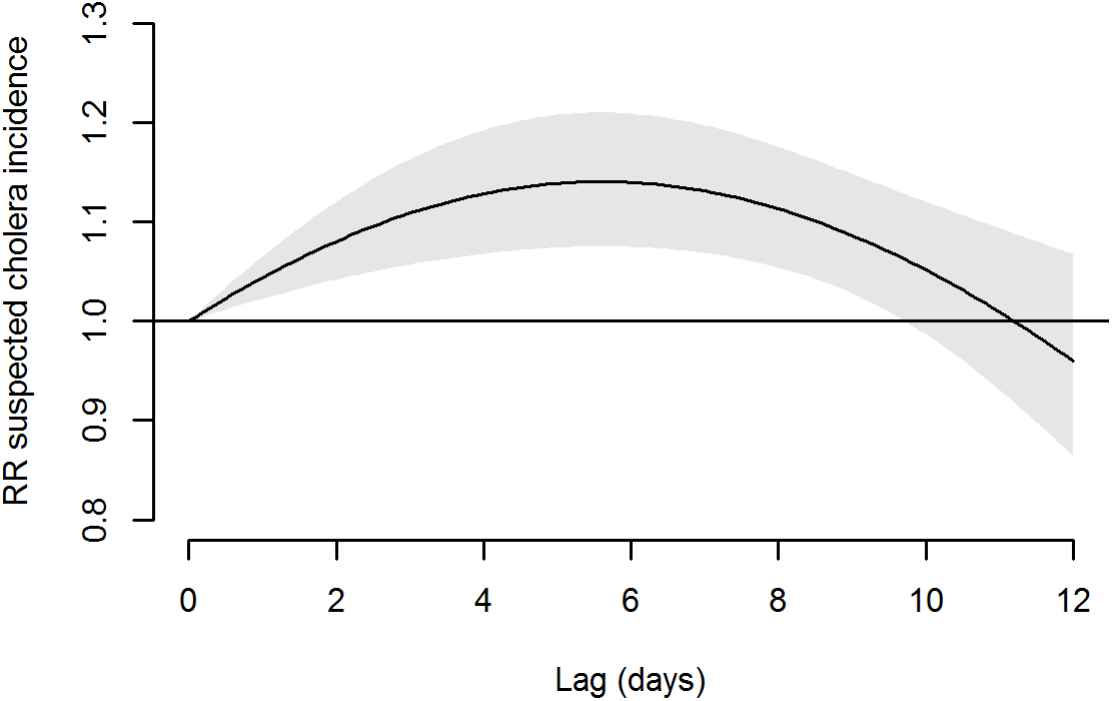
Association of absence of water supply (0 m^3^ of water supplied at day 0) with suspected cholera incidence on the 12 following days. Shaded area: 95% CI of RR.

The temporal distribution of the effects is summarized in Fig 4. This curve indicates a maximum effect 5 to 7 d after the plant fails to produce any water, with the increased risk then vanishing after 12 d.

Admissions to the CTC attributable to a suboptimal volume supplied were 23.2% (95% CI 11.4%–33.2%) with 1,332 attributed cases out of 5,745.

### Stratification by Neighbourhood Access to Tap Water

**Fig 5:**
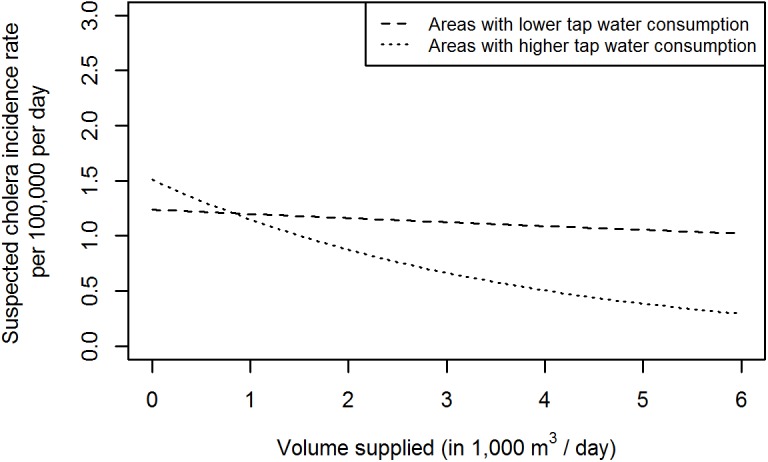
Predicted incidence rate of suspected cholera for 10,000 as a function of water volume supplied, stratified by neighbourhoods with higher (≥2.8 l per person per day) and lower (<2.8 l per person per day) tap water consumption. Model covariates for this prediction were set to the dataset mid-point (September 1, 2011), median precipitation rate value (0.4 mm/h), median number of suspected cholera cases admitted in the other neighbourhood (1 case), and a Thursday.

In the 92 neighbourhoods with a higher tap water consumption, 2,528 CTC admissions were recorded for the study period for an estimated population of 98,000 people (average yearly incidence of 4.8 suspected cholera cases per 1,000), while in the 92 neighbourhoods with a lower tap water consumption, 3,217 suspected cholera cases were admitted for an estimated population of 106,000 inhabitants (average yearly incidence of 5.7 suspected cholera cases per 1,000). Association between predicted incidence rates for suspected cholera in both areas and volume of water supplied by the treatment plant, at set values for other model covariates are shown in Fig 5. Although the incidence is predicted to be generally lower in areas with higher tap water consumption, this incidence increases markedly with a reduction in water availability, whereas the incidence in neighbourhoods with a lower consumption is not influenced by the volume of water produced. The risk of no water produced relative to an optimal production was significantly higher in areas with a higher tap water consumption (RR = 3.71, 95% CI 1.91–7.20) compared to neighbourhoods with a lower consumption (RR = 1.17, 95% CI 0.63–2.14; *p* (z-test) = 0.01).

In the neighbourhoods consuming more tap water, the number of suspected cholera cases attributable to suboptimal water production was estimated to be 793 out of 2,528 (31.4%, 95% CI 17.3–42.5).

### Sensitivity Analysis

Variations of the assumptions on the lag-response function and the flexibility of the cubic spline of date in the main model lead to a 12-day overall cumulative RR of a comparable magnitude, which falls between 2.44 and 6.66 (Table 2). This suggests that the reported results for the cumulative RR are robust to model parameters variation.

**Table 2:** Sensitivity analysis of the effect of the number of df/year in cubic spline of date and lag-response function on the 12-day cumulative association of volume supplied with admissions to CTC.

Model	df/year in cubic spline of date	Lag-response function	12 day cumulative association of absence of water supplied—RR (95% CI)
Main model	12	Polynomial 2nd degree, no intercept	2.55 (1.54–4.24)
Model A	6	Polynomial 2nd degree, no intercept	6.66 (4.31–10.29)
Model B	18	Polynomial 2nd degree, no intercept	2.44 (1.45–4.10)
Model C	12	Polynomial 2nd degree, with intercept	3.08 (1.69–5.60)
Model D	12	Polynomial 3rd degree, no intercept	2.76 (1.60–4.76)
Model E	12	Polynomial 3rd degree, with intercept	2.99 (1.65–5.45)
Model F	12	3 strata with breaks at days 3 and 6	3.15 (1.75–5.69)
Model G	12	5 Strata with breaks at days 2, 4, 6, and 8	3.11 (1.72–5.62)
Model H	12	Polynomial 2nd degree, no intercept*	2.86 (1.47–5.58)

* Model H adjusts for residual chlorine levels in the water supplied, modelled with the same lag structure as volume.

df, degrees of freedom

We found no evidence for confounding of the volume effect by residual chlorine concentration (Model H), which was not surprising given its low correlation with volume (r = -0.03). In fact, there was no evidence in these data for an association between residual chlorine levels in the water supplied and suspected cholera. The RR for low residual chlorine (0.4 mg/l) compared to high (0.8 mg/l) was 0.7 (0.43–1.14), when chlorine was entered into the model with the same 12-day lag structure as used for volume.

## Discussion

Using time-series regression methods to investigate the relationship between chlorinated tap water availability and suspected cholera incidence, our results showed that the unreliability of tap water supply in a cholera endemic setting was associated with a more than 2-fold increase in suspected cholera incidence at city level, and a nearly 4-fold increase in areas with higher tap water consumption. The results also showed that 23% of the cases admitted at the CTC in Uvira between 2009 and spring 2014 may be attributed to irregular tap water supply.

Our study suggests that cholera incidence in Uvira could possibly be reduced by nearly a quarter by ensuring that the existing water treatment infrastructure performed better and supplied water daily in regular and sufficient quantity to its existing customers, even in a town where a single tap serves, on average, nearly 60 people. These results substantiate the increased annual risk of infection due to water supply failure estimated by Hunter and colleagues for other diarrhoeal pathogens by means of Quantitative Microbiological Risk Assessment (QMRA) [21]. These calculations showed a 13% increase in annual risk of infection for rotavirus or Enterotoxigenic *Escherichia coli* after a single day of water supply failure.

Interruptions in piped water supply are likely to increase cholera incidence in an endemic setting through multiple pathways. In the absence of other improved sources of water in Uvira, households can be expected to revert to unsafe water sources when tap water is not available, or they may reduce their use of water and thereby restrict hygiene behaviours that can reduce person-to-person cholera transmission. The unreliability of tap water supply may also encourage households to store large amounts of water for longer periods, leading to an increased risk of water contamination, as was observed in East Africa [22]. In Bangladesh, *Vibrio cholerae* was found in 1.2% of water storage vessels in households that used a safe water source free of contamination [23]. A recent study also showed that stored water collected from improved sources in Cambodia represented a similar risk for health to that from stored water collected from other sources, when comparing microbial quality [24].

The results of this study highlight the need to take into account the reliability of tap water supply when considering it as an “improved” water source. The term “improved” was adopted by the WHO/UNICEF Joint Monitoring Program (JMP) in charge of monitoring progress toward the Millennium Development Goals (MDGs) for water and sanitation, and uses the technology of the household water supply as a proxy for the water’s quality. According to the JMP, improved water sources include piped supplies, protected wells, and boreholes, but exclude surface water sources or unprotected springs. JMP monitoring figures showed that approximately 2.3 billion people have gained access to an improved water source in the last 10 y, while 70% of that number gained access to piped water on premises, although these definitions fail to give any indication on the continuity of the water source used [25]. Recognizing this limitation, the Post-2015 Water Working Group assigned by the JMP to develop new metrics for monitoring global water access, now includes the concept of supply reliability in its definition of a “safely managed” water source. Households having access to an improved source of water “on-premises” that fails, on average, less than 2 d in 2 wk; provides water in sufficient quantity for domestic use; meets WHO guidelines for *E*. *coli*, arsenic, and fluoride; and is subject to a risk-management plan would be considered as using a safely managed water source, while households using an improved water source within 30 min, including queuing, of the house would be considered as having access to basic drinking water [26]. Our study findings support the inclusion of supply reliability in the definition of a higher level of access to drinking water and highlight the health implications of piped water supply unreliability. Our findings suggest that a single day of supply interruption may translate in a significant increase in suspected cholera incidence, especially in neighbourhoods where people rely more heavily on piped water, and the health implications of the JMP threshold of 2 d of failure within a fortnight can be questioned. In addition, the issue of perceived reliability of the supply by users may also be examined, when considering that the health benefits associated with access to piped water supply on premises derive partly from the decreasing need for water storage in the household. Indeed, if households still experience regular unplanned piped supply interruptions, they are likely to keep storing water as a coping strategy for these unexpected cuts, potentially reducing the health benefits expected with piped water on premises. This suggests that avoiding interruptions of piped supplies or mitigating the impact of such interruptions—by informing users ahead of their occurrence when interruptions are planned, or of the expected duration of the interruption when unexpected for example—should be a major element of the risk management plan for these safely managed sources.

Our results also underline the importance of appropriately characterizing water supply used when quantifying the health risk of using “unimproved” water sources. Indeed, the counterfactual often relates to population groups using piped water on premises [27]. These “reference” groups may actually bear an increased health risk linked to the unreliability of the water supply, which remains uncontrolled for in most health impact studies of water interventions. This may explain why an updated review on water-related interventions, published in *The Lancet’s* “Global Burden of Disease” series, found no evidence of a significant diarrhoea risk reduction from access to “on-plot” water supply compared to access to other “improved” sources of water [27].

The presence of residual chlorine in the water fed into the distribution network in Uvira indicates that the chlorination at the plant was sufficient to remove existing microbial contamination from the source of the treatment plant. Due to the complexity of chlorine decay in the distribution system between the treatment plant and the tap, these figures are unlikely representative of the amount of residual chlorine at the taps, although chlorine residuals at the taps can only be lower than those at the treatment plant [28]. These levels of residual chlorine in the water supplied, most of the time comprised between 0.5 and 0.7 mg/l, may not be enough to prevent water contamination in the distribution network, especially after interruptions and at low pressure [29]. Water contamination by consumers during collection and storage may also be a significant means of cholera transmission that could be reduced with higher levels of residual chlorine in the supplied water. Our analysis did not reveal any association between levels of residual chlorine at the treatment plant and suspected cholera incidence, but it did not have the required statistical power to investigate a potential interaction between volume supplied and chlorination in detail.

One limitation of this study is that the case definition used may have included patients admitted to the CTC for diarrhoea of aetiology other than cholera infection. Indeed, CTC admissions were rarely confirmed by laboratory tests. A study on cholera rapid diagnostic tests in DRC found that 73% of suspected cholera cases tested during an outbreak in Katanga province were laboratory-confirmed by culture and PCR [30]. Misclassification of admissions as cholera cases would not pose a threat to the validity of comparisons over time as performed by the present study, except if the proportion of non-cholera cases decreases during higher incidence periods, which cannot be excluded.

Another limitation of this study is the assumption that all cholera cases in Uvira seek health care at the CTC. No evidence is available to confirm or reject this assumption, but little alternative to treat severe diarrhoea and associated dehydration is available in Uvira besides the CTC. Furthermore, the CTC provides health care free of charge, and direct costs of treatment should not act as a factor in socioeconomic selection bias. It is unlikely that the potential bias introduced by using health facility data would vary significantly over time, which is important for the present analysis.

Similarly, this analysis assumes that all cholera cases presenting to the CTC are admitted, even during outbreak periods when the CTC is reaching its capacity limit. However a mechanism has been in place since 2009 to strengthen CTC capacity when the number of admissions exceeds 25 per week. It is therefore reasonable to consider that the CTC did not refuse patients due to limited bed space.

Time-series regression modelling does not directly incorporate variation in immunity in the population though the inclusion as regressor of a cubic spline of time will indirectly allow for such fluctuation, if smooth, so the limitation should be minor. This applies, as well, to other potential unmeasured confounders linked with seasonality or showing smooth variations, such as population nutritional status or disposable income. In particular, we considered that the spline function of time-captured variations of the economic cost and affordability of water, as no sudden official water tariffs changes were recorded during the study period. In addition, price paid for water in Uvira is highly heterogeneous and unregulated due to the sharing of private taps and reselling of tap water. Although power supply interruptions in Uvira can be considered as abrupt and are possibly more frequent during the dry season due to less energy production in hydroelectric dams, power supply interruptions should be considered as a cause of variations of the exposure of interest rather than a potential confounder. In a town where less than 8% of the population reports owning a refrigerator, power supply interruptions are unlikely to be associated with suspected cholera incidence by means of inappropriate food preservation. A moderate overdispersion in model residuals (scale parameter of 1.88) was observed and was accounted for by modelling with a quasi-Poisson family. Assuming that the distribution of daily cases would otherwise follow a Poisson distribution, this overdispersion more generally attests to the model not capturing all of the systematic causes of variation in cholera incidence. However, there seems little reason to expect such causes to be associated with water volume, which would be necessary to cause bias in the relative risks reported. Residual confounding cannot be excluded, but a causal relationship seems the most likely explanation for the observed association. In reference to Bradford Hill’s criteria for causality, our results indeed demonstrate a reasonably strong effect occurring after exposure, with a dose-response pattern and a stronger association in areas with higher tap water consumption, and along a plausible and coherent biological pathway [31].

To conclude, our results showed a clear association between the poor reliability of the water supply system in Uvira and the incidence of suspected cholera in the entire city. They suggest that supply interruptions and unreliability increase population exposure to unsafe sources of water, encourage water storage in households and restrict hygiene practices, all of which translate into increased suspected cholera transmission. They argue in favour of water supply investments that focus on the delivery of a reliable and sustainable water supply and not only on point-of-use water quality improvements as are often seen during cholera outbreaks.

## Supporting Information

S1 Table Dataset. (CSV)

S1 Text Data analysis strategy and timeline. (DOCX)

S2 Text STROBE checklist. (DOC)

S3 Text R script. (R)

## Abbreviations

CI: confidence interval
CTC: cholera treatment centre
df: degree of freedom
DLNM: distributed lag nonlinear models
DPD: N,N diethyl-p-phenylene diamine
DRC: Democratic Republic of the Congo
GPS: global positioning system
JMP: Joint Monitoring Programme
NASA GES DISC: NASA Goddard Earth Sciences Data and Information Services Center
NGO: non-governmental organisation
MDG: Millennium Development Goals
QMRA: Quantitative Microbiological Risk Assessment
RR: relative risk
WHO: World Health Organization
