# Supplementary material for: Water Supply Interruptions and Suspected Cholera Incidence: A Time-Series Regression in the Democratic Republic of the Congo
Source: PLoS Med. 2015 Oct 27;12(10):e1001893. doi: 10.1371/journal.pmed.1001893 (PMC4624412; doi:10.1371/journal.pmed.1001893)
Supplement: S1 Text — (DOCX) [file pmed.1001893.s002.docx]

**Water supply interruptions and endemic suspected cholera incidence: a time-series regression**

**Data analysis timeline**

January 2014: Compilation of suspected cholera admissions and treatment plant production dataset obtained from Uvira CTC and Regideso, with data entry quality checks against scanned paper records.

February 2014: Compilation of the remote sensing data for precipitation rates in Uvira from NASA Tropical Rainfall Measuring Mission (TRMM) 3B42-v7, through the Giovanni online data system

February 2014: First linear lag and DLNM modelling of the data for the entire city, excluding residual chlorine levels, with sensitivity analysis of model parameters

March 2014: LSHTM Ethics Committee approval

June 2014: Extension of the dataset to 30^th^ of April 2014, obtained from Uvira CTC and Regideso. Stratification of the dataset by areas with higher or lower tap water consumption, based on Regideso tap meters mapping and Regideso invoicing information in February 2012

July-August 2014: Modelling of the extended dataset, including interaction between volume produced and residual chlorine levels and stratification by tap water consumption.

April 2015: In response to Plos Medicine reviewers, correction of the inclusion of residual chlorine levels in the model and revisions of the sensitivity analysis. Inclusion in the manuscript of the results from the stratification of town by tap water consumption.
