# Supplementary material for: Water Supply Interruptions and Suspected Cholera Incidence: A Time-Series Regression in the Democratic Republic of the Congo
Source: PLoS Med. 2015 Oct 27;12(10):e1001893. doi: 10.1371/journal.pmed.1001893 (PMC4624412; doi:10.1371/journal.pmed.1001893)
Supplement: S2 Text — (DOC) [file pmed.1001893.s003.doc]

**STROBE Statement—checklist of items that should be included in reports of observational studies**

**Manuscript submitted to Plos Medicine entitled “Water supply interruptions and endemic cholera incidence: a time-series regression” (PMEDICINE-D-15-00215R1)**

|  | Item No | Recommendation | Manuscript excerpt |
| --- | --- | --- | --- |
| **Title and abstract** | 1 | (*a*) Indicate the study’s design with a commonly used term in the title or the abstract | Title: “Water supply interruptions and endemic cholera incidence: a time-series regression” |
|  |  | (*b*) Provide in the abstract an informative and balanced summary of what was done and what was found | Abstract: “Methods and findings: Time-series patterns of daily admissions to the Cholera Treatment Centre in Uvira in South Kivu Province between 2009 and 2014 were examined in relation to the daily variations in volume of water supplied by the town water treatment plant. Quasi-poisson regression and distributed lag non-linear models up to 12 days were used, adjusting for daily rainfall, day of the week and seasonal variations. A total of 5,745 patients over 5 years of age were admitted to the CTC over the study period of 1,946 days. Following a day without tap water supply, the cholera incidence rate increased on average by 155% over the next 12 days, corresponding to a rate ratio of 2.55 (95% CI: 1.54 – 4.24), compared to the incidence experienced after a day with optimal production (defined as the 95th percentile - 4,794 m3). Cases attributable to a sub-optimal tap water supply reached 23.2% of total cases (95% CI 11.4% - 33.2%). Although generally reporting less admissions to the CTC, neighbourhoods with a higher access to tap water were more affected by water supply interruptions, with a rate ratio of 3.71 (95% CI: 1.91 – 7.20) and an attributable fraction of cases of 31.4% (95% CI: 17.3% – 42.5%). The analysis did not suggest any association between levels of residual chlorine in the water fed to the distribution network and cholera incidence. Laboratory confirmation of cholera was not available for this analysis.” |
| Introduction | | |  |
| Background/rationale | 2 | Explain the scientific background and rationale for the investigation being reported | Introduction: “…The degree to which the unreliability of clean water supply affects cholera incidence, especially in endemic areas, has not been previously investigated.” |
| Objectives | 3 | State specific objectives, including any prespecified hypotheses | End of Introduction: “this study explores the association between the daily volume of chlorinated tap water distributed to households and admissions to the local Cholera Treatment Centre (CTC) with a time-series regression analysis” |
| Methods | | |  |
| Study design | 4 | Present key elements of study design early in the paper | End of Introduction: “this study explores the association between the daily volume of chlorinated tap water distributed to households and admissions to the local Cholera Treatment Centre (CTC) with a time-series regression analysis” |
| Setting | 5 | Describe the setting, locations, and relevant dates, including periods of recruitment, exposure, follow-up, and data collection | All Methods section |
| Participants | 6 | (*a*) *Cohort study*—Give the eligibility criteria, and the sources and methods of selection of participants. Describe methods of follow-up  *Case-control study*—Give the eligibility criteria, and the sources and methods of case ascertainment and control selection. Give the rationale for the choice of cases and controls  *Cross-sectional study*—Give the eligibility criteria, and the sources and methods of selection of participants | Methods section, “Suspected cholera cases and water supply data”: “Daily admissions to the CTC during the period 1st of January 2009 to 30th of April 2014 were extracted from the database held by the district health office since 2009, which is updated weekly from the paper register used by clinical staff at the CTC. Cases are recorded along with their neighbourhood of residence. In order to conform to the WHO cholera case definition in endemic areas, patients aged 5 years or under were excluded [9].” |
|  |  | (*b*) *Cohort study*—For matched studies, give matching criteria and number of exposed and unexposed  *Case-control study*—For matched studies, give matching criteria and the number of controls per case | NA |
| Variables | 7 | Clearly define all outcomes, exposures, predictors, potential confounders, and effect modifiers. Give diagnostic criteria, if applicable | Methods section, all “Statistical methods” paragraph: “The relationship between the daily number of admissions at the CTC and the daily volume of water supplied by the Regideso was examined using generalized linear Poisson regression models allowing for overdispersion [15]. To account for seasonality and long-term trends in potential unmeasured confounders, a cubic spline of date (12 degrees of freedom [df] per year) was included in the model. Terms for the day of the week (indicators) and daily rainfall (linear) were also added. An autoregressive term was introduced to the model in order to control for a significant temporal auto-correlation at days 1 and 2.  The relationship between water supply level and cholera incidence was modelled using the framework of distributed lag non-linear models (DLNMs). These models allow the net effect of an exposure to be computed as the sum of contributions at different lags, through the definition of a lag-response curve in addition to the exposure-response relationship [16]. DLNMs were originally developed for investigating temperature-health associations [17].  Informed by a recent review of incubation periods for cholera and delays due to storage capacity in the water production system, we considered lags up to 12 days and assumed that effects, if any, rose from none at lag 0 [18].  A linear exposure-response function scaled against the 95th percentile of volume (subsequently referred to as optimal production) was considered for association between volume and admissions. The delay in effect of water volume on admissions (modelled as a lag-response curve) was constrained to follow a smooth quadratic curve.  The overall cumulative effect of volume produced on CTC admissions over 12 days was computed by summing effects over the whole lag period. The association is reported as relative risk (RR).  The number and fraction of cases attributable to water production lower than the 95th percentile of volume were estimated from the model, as defined by Gasparrini and Leone [19]. These calculations assume that a fraction (RR-1)/RR of cholera cases on any given day is attributable to the exposure to a sub-optimal water supply during the 12 previous days modelled to give a relative risk RR, and are computed for the entire study duration. The figures are interpreted as the number of cases attributable to sub-optimal water production during the study period, and potentially preventable if the volume of water supplied was maintained above the optimal production level.  We explored the potential heterogeneity of effect in neighbourhoods with a higher or a lower access to tap water. The median value of tap water invoiced across neighbourhood in February 2012 was 2.8l per person and per day and this was taken as cut-off value for stratification of neighbourhoods into higher and lower access to tap water. A regression model was fitted separately to each series of higher and lower access, based on the same specifications used in the main analysis. These models also included the number of admissions in the other area as an additional term, in order to account for potential inter-neighbourhoods transmission.  The sensitivity of the estimates to the modelling assumptions outlined above was tested by the comparison with models specified with different choices.” |
| Data sources/ measurement | 8* | For each variable of interest, give sources of data and details of methods of assessment (measurement). Describe comparability of assessment methods if there is more than one group | Methods section, all “Suspected cholera cases and water supply data” paragraph: “Daily admissions to the CTC during the period 1st of January 2009 to 30th of April 2014 were extracted from the database held by the district health office since 2009, which is updated weekly from the paper register used by clinical staff at the CTC. Cases are recorded along with their neighbourhood of residence. In order to conform to the WHO cholera case definition in endemic areas, patients aged 5 years or under were excluded [9].  Daily volume of water supplied for the same time period was collected from the register held at the Regideso water treatment plant in Uvira. Volumes of water supplied over 24 hours were measured with a flowmeter placed at the only water treatment station output, where water is pumped to a single 1’600 m3 reservoir feeding the town gravity distribution system. At the same station output, the levels of residual chlorine in the supplied water were measured by means of DPD (N,N diethyl-p-phenylene diamine) tablets and the daily average over 2 or more measures recorded in the same register.  The 184 neighbourhoods of Uvira were classified as having a higher or lower level of access to tap water based on an estimated monthly average volume of tap water available per person. This was calculated for each neighbourhood on the basis of the volume of water billed in February 2012 to 2,630 Regideso taps (each of which is geographically referenced and allocated to a particular neighbourhood by GIS) divided by the estimated neighbourhood population. Based on actual tap metering, this estimate accounts for the large variability between taps in water use (shared or private) and water availability that is dependent on the tap location on the distribution network.  Daily rainfall estimates averaged for Uvira area at a 0.25 degree by 0.25 degree spatial resolution were obtained from NASA Tropical Rainfall Measuring Mission (TRMM) 3B42-v7, through the Giovanni online data system, developed and maintained by the NASA Goddard Earth Sciences Data and Information Services Center (NASA GES DISC). Daily variations of temperature are small in Uvira, and no complete daily temperature record for the region was identified. Temperature was therefore not included as a confounder in the analysis.” |
| Bias | 9 | Describe any efforts to address potential sources of bias | Discussion section, limitations: “One limitation of this study is that the case definition used may have included patients admitted to the CTC for diarrhoea of aetiology other than cholera infection. Indeed cases were rarely confirmed by laboratory tests. A study on cholera rapid diagnostic tests in DRC found that, 73% of cases tested during an outbreak in Katanga province were laboratory confirmed by culture and PCR [29]. Misclassification of admissions as cholera cases would not pose a threat to the validity of comparisons over time as performed by the present study, except if the proportion of non-cholera cases decreases during higher incidence periods, which cannot be excluded.  Another limitation of this study is the assumption that all cholera cases in Uvira seek healthcare at the CTC. No evidence is available to confirm or reject this assumption, but little alternative to treat severe diarrhoea and associated dehydration is available in Uvira besides the CTC. Furthermore, the CTC provides healthcare free of charge, and direct costs of treatment should not act as a factor in socio-economic selection bias. It is unlikely that the potential bias introduced by using health facility data would vary significantly over time, which is important for the present analysis.  Similarly this analysis assumes that all cholera cases presenting to the CTC are admitted, even during outbreak periods when the CTC is reaching its capacity limit. However a mechanism has been in place since 2009 to strengthen CTC capacity when the number of cases exceeds 25 cases per week. It is therefore reasonable to consider that the CTC did not refuse patients due to overcapacity.  Time-series regression modelling does not directly incorporate variation in immunity in the population though the inclusion as regressor of the smooth function of time will indirectly allow for such fluctuation, if smooth, so the limitation should be minor. This applies as well to other potential unmeasured confounders linked with seasonality or showing smooth variations, such as population nutritional status or economic cost of water. A moderate overdispersion in model residuals (scale parameter of 1•88) is observed, and was accounted for by modelling with a quasi-Poisson family. Assuming that the distribution of daily cases would otherwise follow a Poisson distribution, this overdispersion more generally attests to the model not capturing all of the systematic causes of variation in cholera incidence. However, there seems little reason to expect such causes to be associated with water volume, which would be necessary to cause bias in the relative risks reported. Residual confounding cannot be excluded, but a causal relationship seems the most likely explanation for the observed association.” |
| Study size | 10 | Explain how the study size was arrived at | NA |
| Quantitative variables | 11 | Explain how quantitative variables were handled in the analyses. If applicable, describe which groupings were chosen and why | Methods section, “Statistical Methods” paragraph: “The relationship between the daily number of admissions at the CTC and the daily volume of water supplied by the Regideso was examined using generalized linear Poisson regression models allowing for overdispersion [15]. To account for seasonality and long-term trends in potential unmeasured confounders, a cubic spline of date (12 degrees of freedom [df] per year) was included in the model. Terms for the day of the week (indicators) and daily rainfall (linear) were also added. An autoregressive term was introduced to the model in order to control for a significant temporal auto-correlation at days 1 and 2.” “Informed by a recent review of incubation periods for cholera and delays due to storage capacity in the water production system, we considered lags up to 12 days and assumed that effects, if any, rose from none at lag 0 [18].  A linear exposure-response function scaled against the 95th percentile of volume (subsequently referred to as optimal production) was considered for association between volume and admissions. The delay in effect of water volume on admissions (modelled as a lag-response curve) was constrained to follow a smooth quadratic curve.” |
| Statistical methods | 12 | (*a*) Describe all statistical methods, including those used to control for confounding | Methods section, all “Statistical methods” paragraph |
|  |  | (*b*) Describe any methods used to examine subgroups and interactions | Methods section: “We explored the potential heterogeneity of effect in neighbourhoods with a higher or a lower access to tap water. The median value of tap water invoiced across neighbourhood in February 2012 was 2.8l per person and per day and this was taken as cut-off value for stratification of neighbourhoods into higher and lower access to tap water. A regression model was fitted separately to each series of higher and lower access, based on the same specifications used in the main analysis. These models also included the number of admissions in the other area as an additional term, in order to account for potential inter-neighbourhoods transmission.” |
|  |  | (*c*) Explain how missing data were addressed | NA |
|  |  | (*d*) *Cohort study*—If applicable, explain how loss to follow-up was addressed  *Case-control study*—If applicable, explain how matching of cases and controls was addressed  *Cross-sectional study*—If applicable, describe analytical methods taking account of sampling strategy | NA |
|  |  | (*e*) Describe any sensitivity analyses | Methods section: “The sensitivity of the estimates to the modelling assumptions outlined above was tested by the comparison with models specified with different choices.” |

Continued on next page

| Results | | |  |
| --- | --- | --- | --- |
| Participants | 13* | (a) Report numbers of individuals at each stage of study—eg numbers potentially eligible, examined for eligibility, confirmed eligible, included in the study, completing follow-up, and analysed | Results section: “Between January 1st, 2009 and April 30th, 2014, a total of 5,745 patients aged over 5 years were admitted to the CTC, with daily admissions ranging between 0 and 32. Daily volume of water supplied to the city ranged from 0 to 5,748 m3 with a 95th percentile (“optimal” production) of 4,794 m3, while the level of residual chlorine ranged from 0.1 to 1.7 mg/l (Table 1 & Figure 1).” |
|  |  | (b) Give reasons for non-participation at each stage | NA |
|  |  | (c) Consider use of a flow diagram | NA |
| Descriptive data | 14* | (a) Give characteristics of study participants (eg demographic, clinical, social) and information on exposures and potential confounders | Results section: “Between January 1st, 2009 and April 30th, 2014, a total of 5,745 patients aged over 5 years were admitted to the CTC, with daily admissions ranging between 0 and 32. Daily volume of water supplied to the city ranged from 0 to 5,748 m3 with a 95th percentile (“optimal” production) of 4,794 m3, while the level of residual chlorine ranged from 0.1 to 1.7 mg/l (Table 1 & Figure 1).” |
|  |  | (b) Indicate number of participants with missing data for each variable of interest | Table 1 |
|  |  | (c) *Cohort study*—Summarise follow-up time (eg, average and total amount) | Results section: “Between January 1st, 2009 and April 30th, 2014, a total of 5,745 patients aged over 5 years were admitted to the CTC, with daily admissions ranging between 0 and 32. Daily volume of water supplied to the city ranged from 0 to 5,748 m3 with a 95th percentile (“optimal” production) of 4,794 m3, while the level of residual chlorine ranged from 0.1 to 1.7 mg/l (Table 1 & Figure 1).” |
| Outcome data | 15* | *Cohort study*—Report numbers of outcome events or summary measures over time | Results section: “Between January 1st, 2009 and April 30th, 2014, a total of 5,745 patients aged over 5 years were admitted to the CTC, with daily admissions ranging between 0 and 32. Daily volume of water supplied to the city ranged from 0 to 5,748 m3 with a 95th percentile (“optimal” production) of 4,794 m3, while the level of residual chlorine ranged from 0.1 to 1.7 mg/l (Table 1 & Figure 1).” |
|  |  | *Case-control study—*Report numbers in each exposure category, or summary measures of exposure |  |
|  |  | *Cross-sectional study—*Report numbers of outcome events or summary measures |  |
| Main results | 16 | (*a*) Give unadjusted estimates and, if applicable, confounder-adjusted estimates and their precision (eg, 95% confidence interval). Make clear which confounders were adjusted for and why they were included | Results section: “Figure 3 displays the overall cumulative exposure-response relationship between volume of water supplied and incidence of cholera as modelled with the DLNM time-series regression. When the plant produces no water, the model predicts an increase of 155% in the number of cholera cases within the next 12 days, corresponding to an RR of 2.55 (95%CI: 1.54 - 4.24), compared to the optimal production level (4,794 m3).” |
|  |  | (*b*) Report category boundaries when continuous variables were categorized | NA |
|  |  | (*c*) If relevant, consider translating estimates of relative risk into absolute risk for a meaningful time period | NA |
| Other analyses | 17 | Report other analyses done—eg analyses of subgroups and interactions, and sensitivity analyses | Results section: “Stratification by neighbourhood access to tap water  In the 92 neighbourhoods with a better access to tap water, 2,528 cases were recorded for the study period for an estimated population of 98,000 people (average yearly incidence of 4.8 cases per 1,000), while in the 92 neighbourhoods with a poorer access to tap water, 3,217 cases were admitted for an estimated population of 106,000 inhabitants (average yearly incidence of 5.7 cases per 1,000). Although the incidence was generally lower in areas with better access to tap water, the risk of no water produced relative to an optimal production was significantly higher in those areas (RR = 3.71, 95% CI 1.91 – 7.20) compared to neighbourhoods with a poorer access (RR = 1.17, 95% CI 0.63 – 2.14; p (z-test) = 0.01). The two overall cumulative exposure-response relationships are shown in Figure 5.  Figure 5: 12-day cumulative association of volume of tap water supplied with cholera incidence, stratified by neighbourhoods with higher and lower access to tap water in Uvira (patterned areas: 95% CI of RR)  In the neighbourhoods better served by the Regideso distribution system, the number of cases attributable to sub-optimal water production was estimated to be 793 cases out of 2,528 (31.4%, 95% CI 17.3 – 42.5).  Sensitivity analysis  Variations of the assumptions on the lag-response function and the flexibility of the cubic spline of date in the main model lead to a 12-day overall cumulative RR of a comparable magnitude (Table 2).” + Table 2 |
| Discussion | | |  |
| Key results | 18 | Summarise key results with reference to study objectives | Discussion section: “Using time-series regression methods to investigate the relationship between chlorinated tap water availability and cholera incidence, our results showed that the unreliability of tap water supply in a cholera endemic setting can lead to a more than two-fold increase in cholera incidence at city level, and to a nearly four-fold increase in areas with a better access to tap water. The results also showed that nearly a quarter of the cholera cases admitted in Uvira between 2009 and spring 2014 could be attributed to irregular tap water supply.” |
| Limitations | 19 | Discuss limitations of the study, taking into account sources of potential bias or imprecision. Discuss both direction and magnitude of any potential bias | Discussion section: “Our analysis did not reveal any association between levels of residual chlorine at the treatment plant and cholera incidence, but it did not have the required statistical power to investigate a potential interaction between volume supplied and chlorination in detail.  One limitation of this study is that the case definition used may have included patients admitted to the CTC for diarrhoea of aetiology other than cholera infection. Indeed cases were rarely confirmed by laboratory tests. A study on cholera rapid diagnostic tests in DRC found that, 73% of cases tested during an outbreak in Katanga province were laboratory confirmed by culture and PCR [29]. Misclassification of admissions as cholera cases would not pose a threat to the validity of comparisons over time as performed by the present study, except if the proportion of non-cholera cases decreases during higher incidence periods, which cannot be excluded.  Another limitation of this study is the assumption that all cholera cases in Uvira seek healthcare at the CTC. No evidence is available to confirm or reject this assumption, but little alternative to treat severe diarrhoea and associated dehydration is available in Uvira besides the CTC. Furthermore, the CTC provides healthcare free of charge, and direct costs of treatment should not act as a factor in socio-economic selection bias. It is unlikely that the potential bias introduced by using health facility data would vary significantly over time, which is important for the present analysis.  Similarly this analysis assumes that all cholera cases presenting to the CTC are admitted, even during outbreak periods when the CTC is reaching its capacity limit. However a mechanism has been in place since 2009 to strengthen CTC capacity when the number of cases exceeds 25 cases per week. It is therefore reasonable to consider that the CTC did not refuse patients due to overcapacity.  Time-series regression modelling does not directly incorporate variation in immunity in the population though the inclusion as regressor of the smooth function of time will indirectly allow for such fluctuation, if smooth, so the limitation should be minor. This applies as well to other potential unmeasured confounders linked with seasonality or showing smooth variations, such as population nutritional status or economic cost of water. A moderate overdispersion in model residuals (scale parameter of 1•88) is observed, and was accounted for by modelling with a quasi-Poisson family. Assuming that the distribution of daily cases would otherwise follow a Poisson distribution, this overdispersion more generally attests to the model not capturing all of the systematic causes of variation in cholera incidence. However, there seems little reason to expect such causes to be associated with water volume, which would be necessary to cause bias in the relative risks reported. Residual confounding cannot be excluded, but a causal relationship seems the most likely explanation for the observed association.” |
| Interpretation | 20 | Give a cautious overall interpretation of results considering objectives, limitations, multiplicity of analyses, results from similar studies, and other relevant evidence | All discussion section |
| Generalisability | 21 | Discuss the generalisability (external validity) of the study results | All discussion section |
| Other information | | |  |
| Funding | 22 | Give the source of funding and the role of the funders for the present study and, if applicable, for the original study on which the present article is based | Acknowledgements section: “This study was funded by the Agence Française de Développement. The funders had no role in study design, data collection and analysis, decision to publish, or preparation of the manuscript.” |

*Give information separately for cases and controls in case-control studies and, if applicable, for exposed and unexposed groups in cohort and cross-sectional studies.

**Note:** An Explanation and Elaboration article discusses each checklist item and gives methodological background and published examples of transparent reporting. The STROBE checklist is best used in conjunction with this article (freely available on the Web sites of PLoS Medicine at http://www.plosmedicine.org/, Annals of Internal Medicine at http://www.annals.org/, and Epidemiology at http://www.epidem.com/). Information on the STROBE Initiative is available at www.strobe-statement.org.
